# Connection between Celiac Disease and Systemic Lupus Erythematosus in Children—A Development Model of Autoimmune Diseases Starting from What We Inherit to What We Eat

**DOI:** 10.3390/nu15112535

**Published:** 2023-05-29

**Authors:** Vasile Valeriu Lupu, Elena Jechel, Cristina Maria Mihai, Elena Cristina Mitrofan, Ancuta Lupu, Iuliana Magdalena Starcea, Silvia Fotea, Adriana Mocanu, Dragos Catalin Ghica, Costica Mitrofan, Dragos Munteanu, Delia Lidia Salaru, Ionela Daniela Morariu, Ileana Ioniuc

**Affiliations:** 1 Faculty of General Medicine, “Grigore T. Popa” University of Medicine and Pharmacy, 700115 Iasi, Romania; 2 Faculty of General Medicine, Ovidius University, 900470 Constanta, Romania; 3 CF Clinical Hospital, 700506 Iasi, Romania; 4 Clinical Medical Department, Faculty of Medicine and Pharmacy, “Dunarea de Jos” University of Galati, 800008 Galati, Romania; 5 Faculty of Pharmacy, “Grigore T. Popa” University of Medicine and Pharmacy, 700115 Iasi, Romania

**Keywords:** celiac disease, systemic lupus erythematosus, children, nutrition, microbiome

## Abstract

Celiac disease (CD) and systemic lupus erythematosus (SLE) are two diseases intensively studied in all age groups, with an increasing incidence at the global level, possibly due to the increased awareness of the diseases and their accurate diagnosis and as a consequence of the new research and innovation technologies that have appeared in medicine. The first is a controllable condition found in approximately 1% of the entire population in the form of a reaction to environmental stimuli affecting individuals with genetic susceptibility, causing gluten intolerance, gastrointestinal and extradigestive symptoms, starting from subclinical stages and culminating in severe malabsorption. On the other hand, lupus is an autoimmune disease with chameleon-like symptoms and found mainly in the female sex, which leaves its clinical mark on most organs, from the skin, eyes, and kidneys to the cardiovascular, pulmonary, neurological, osteoarticular, and hematological systems. Current studies focus on the correlation between celiac disease and other autoimmune pathologies such as autoimmune thyroiditis (Hashimoto and Graves–Basedow), type I diabetes, and systemic lupus erythematosus. The current review aims to present a summary of the data from the specialized literature regarding the intercurrents between celiac disease and lupus by analyzing the most recent studies published on PubMed.

## 1. Introduction

Celiac disease, also called gluten-sensitive enteropathy, is characterized by a disturbance of the internal environment associated with histological changes in the small intestine, the most important of which is the subtotal atrophy of the villi with hyperplasia of the crypts. Clinically, it is manifested by a wide spectrum of symptoms, from gastrointestinal disorders (diarrhea, bloating, weight loss, and abdominal pain) to extra-intestinal symptoms (iron deficiency anemia, delayed puberty, and oral ulcers), centered on variable degrees of malabsorption [1,2]. With an estimated prevalence of 1:100, serological screening is performed by titrating anti-tissue transglutaminase antibodies (TGAs) considered positive at values over two times the normal limit, doubled by genetic testing for human leukocyte antigen (HLA)-DQ2 or HLA-DQ8 and total immunoglobulin A dosing. Regarding duodenal biopsy, the European Society for Paediatric Gastroenterology, Hepatology and Nutrition (ESPGHAN) recommends its use in cases with positive TGA-IgA < 10 times the upper limit of normal values, with the possibility of abstaining if TGA-IgA is over 10 × the upper limit of normal values, in association with the positivity of endomysial antibodies in two blood samples. In the literature, however, there are authors adept at confirming the diagnosis through digestive endoscopy with biopsy in those with equivocal screening results but with strong clinical suspicion after a period of time in which the child follows a normal diet (with gluten content) [2,3,4,5].

Juvenile systemic lupus erythematosus is a multisystemic autoimmune/inflammatory disease that usually starts before the age of 18 (in approximately 15–25% of all SLE patients) where, unlike the adult form, it shows increased activity depending on aspects such as gender, ethnicity, and the age of onset, which results in significant damage and requires aggressive therapy. The incidence varies between 0.36 and 2.5 per 100,000 children, with a prevalence of 1.89–34.1 per 100,000, a peak incidence around the age of 12.6 years, and a physiopathological basis centered on the involvement of the genetic component, with >7% of patients developing the disease as a result of single mutations, while 5-year survival has improved from 30–40% in the 1950s to >90% in the 1980s. The diagnosis and therapy can be complex, being influenced by the polymorphism of the injuries (more aggressive in terms of the renal, hematological, and neuropsychic components), the need for individualized treatment, and drug interactions or associated comorbidities that can, in some cases, endanger lives. Also worth mentioning are the possible atypical forms of lupus, characterized by the absence of autoantibodies, a severe evolution, and a reserved prognosis, especially under the age of 5 [6,7,8].

Consisting of two well-represented entities in pediatric practice, the CD-SLE correlation represents a crossroads in the study of autoimmune diseases, both in terms of their onset as well as their development, overlap, and treatment. The present narrative review aims to present a summary of data from the specialized literature regarding the relationship between celiac disease and lupus by analyzing the most recent studies published on PubMed.

## 2. Epidemiology

Celiac disease is associated with several autoimmune diseases, of which thyroid disease and type 1 diabetes are defined as “associated conditions” or conditions with increased prevalence but not directly related to gluten ingestion. Loci in the HLA region common to those identified in SLE have been observed, SLE being among the top three autoimmune diseases developed by first-degree relatives of patients with celiac disease. In addition, there is evidence of non-celiac autoimmune diseases in the spouses of patients with celiac disease, which contradicts the claim that the involvement of genetics is the only predisposing cause, since the partners do not share genetic characteristics with each other, but only environmental factors and possibly the microbiome, with an impact on the risk of developing autoimmunity [9,10]. About 30% of all patients with celiac disease have one or more autoimmune conditions, while in the general population, there is a prevalence of 3% to 9.4%. Currently, the available evidence suggests that the common genetic background is the main factor that determines the high prevalence of the association, but it is not clarified whether extrinsic factors related to gluten, such as age at first introduction, concomitant breastfeeding, duration of exposure to gluten and gluten-free diet, influence the link between celiac disease and autoimmune diseases [9].

The importance of effective screening among patients with SLE in order to detect CD was debated in a study carried out in the Middle East, the results showing the increase in prevalence among patients with autoimmune diseases, with a higher correlation in the groups of patients with SLE versus controls. Although the frequency of celiac disease markers is considered to be high in SLE patients, only anti-endomysial (EMA) and TGA showed significant differences compared to controls (data present in the literature). The present study still found that 9.6% of the subjects tested positive for anti-gliadin antibodies (either AGAG or AGAA), 3.5% for TGA and 2.6% for EMA. The negative impact of the lack of screening resides in the possibility of incorrect diagnosis and management, which can lead to unnecessary exposure to immunosuppressive drugs associated with side effects [11].

In support of the previously stated findings, another study carried out at Colentina Hospital and the Institute for Mother and Child Care, Bucharest, Romania, demonstrated the increase in the prevalence of gluten-induced autoimmunity among patients with SLE compared to the general population, identified for TGA-IgA, but not for EMA [12].

## 3. Clinical and Paraclinical Aspects

SLE and CD are two complex diseases, encountered in all age groups (including in pediatric practice), with diverse clinical manifestations involving multiple organ systems and an evolution marked by relapses and remissions, dependent on both environmental factors and individual response to therapy.

The pathogenesis of CD can be attributed to a combination of inflammation, nutrient deficiency caused by malabsorption, and enzyme-mediated autoimmune response. The clinical picture provides an evolutionary description over the years, starting from presentation in the form of diarrhea with malabsorption syndrome and reaching that is nowadays a systemic disease with a serious clinical and histological picture. It can appear at any age and is not limited to the digestive tract, involving almost every organ, including the nervous system, liver, skin, reproductive system, cardiovascular system, and musculoskeletal system. It is usually associated with a more severe clinical and histological picture [13,14].

Regarding the symptoms reported by pediatric age groups, the following were identified, in this order:

- very young children (under 3 years old): diarrhea, delay in physical development, abdominal distention; asymptomatic—6.8%
- preschoolers (3–6 years): abdominal pain, iron deficiency, delay in physical development; asymptomatic—18.9%
- school age (≥6 years): abdominal pain, delay in physical development, diarrhea; asymptomatic—23.7% [13]

As a multisystemic disease with a variable clinical picture in terms of severity, SLE in pre-pubescent children rarely shows positivity of anti-nuclear antibodies (ANAs) at diagnosis, along with a smaller titer of antibodies against double quaternary DNA (anti-dsDNA), and fewer renal and musculoskeletal manifestations. However, there is a greater neuropsychiatric, hematological, and complement system involvement (hypocomplementemia) in contrast to SLE in adolescents, which tends to present as an adult form [15].

The most frequent clinical manifestations described in the literature are represented by constitutional symptoms (fever, fatigue, weight loss), skin damage (malar rash, oral ulcers, vasculitic eruptions, photosensitivity, alopecia, discoid lesions, Raynaud’s phenomenon), muscle-skeletal (often symmetrical, non-erosive polyarthritis of large and small joints, myalgia, rarely myositis), hematological (autoimmune thrombocytopenia, idiopathic thrombocytopenic purpura, leukopenia, granulocytopenia, positive Combs test), cardiac (pericarditis, myocarditis, valvular damage, coronary damage due to arteritis or arteriosclerosis), neuropsychological (from headaches, memory loss to global cerebral dysfunction manifested by paralysis or convulsions), pulmonary (pleurisy, pneumonia, pneumothorax, diffuse interstitial damage, hypertension, and pulmonary hemorrhage) and renal (lupus nephritis, classified in six stages, starting from the mild form and culminating in end-stage renal disease) [16].

Regarding the diagnosis of the two conditions, Table 1 presents the main investigations used, together with the criteria that must be met to certify their positivity.

SLE and CD therefore share common systemic manifestations such as the production of autoantibodies, multiple inflammation, and the deposition of immune complexes. Worthy of emphasis are also the involvement of immune complexes and inflammation in the pathogenesis of the two diseases.

Dermatitis herpetiformis (DH) is another autoimmune manifestation encountered in the form of pruritic vesicles (appearing especially on the elbows, forearms, buttocks, knees, and scalp) associated with gluten-sensitive enteropathy. From an immunological point of view, this is described as the coexistence of aggregates of granular or, to a lesser extent, linear deposits of IgA type (predominantly IgA1, rarely IgA2) at the level of the dermo-epidermal junction and circulating immune complexes containing IgA, in the absence circulating IgA antibodies directed against dermal structures. It was also highlighted with the help of immunoabsorption that the complexes found in DH and CD formed on the basis of the two immune fractions (IgA1 and IgA2) that circulate separately, at the same time identifying different properties and the absence of pathogenic involvement, unlike SLE [24,25,26,27].

Inflammation is also a common characteristic of the two conditions and is found in particular in the composition of each in the form of a pro-inflammatory intestinal environment, which leads to an expansion of gliadin-specific T cells (in people with genetic sensitivity) and the perpetuation of a pro-inflammatory phenotype in the case of CD, or, on the contrary, of a vascular inflammation mediated by IL-6 found in SLE, which has as a consequence the appearance of perivascular and vascular leukocyte infiltration and vascular dysfunction with the implicit increase in cardiovascular mortality [28,29].

## 4. Pathogenic Correlation

Innate immunity represents the first line of defense, determining a rapid response that occurs a few minutes after infection. It is non-specific and does not confer immune memory, being based on the complement system of myeloid cells (neutrophils, monocytes, dendritic cells, and macrophages), natural killer cells (NKs). or innate lymphoid cells (ILCs) responsible for molecular recognition and antigen presentation, phagocytosis, and elimination of pathogens. In contrast, adaptive immunity is provided by B and T lymphocytes and has been described as slow and specific. The origin and development of autoimmune diseases are mainly attributed to an excessive and sustained response to autoantigens mediated by B and T cells. It is well known that autoinflammation driven by dysregulation of innate immune signaling contributes to the establishment of adaptive immune responses that lead to the development of autoimmunity [30,31]. Women have a stronger immune response to infections and vaccination than men, an aspect from which arises their increased susceptibility to the development of autoimmune diseases, possibly because of the influence of sex hormones on the activity of the immune system [32]. Numerous theories highlight that metabolic modification and epigenetic reprogramming may offer potential treatments for chronic inflammation associated with autoimmunity, allowing the balance between hyperinflammation and immunosuppression to be restored and therapeutic benefits to be obtained at the cost of toxicity, reduced bioavailability, and immune-related adverse effects [30].

CD and SLE are two conditions known to be interconnected, and their association has been intensively studied by dosing anti-transglutaminase and anti-endomysial antibodies, followed by duodenal biopsy performed on those with positive IgA-TGA/EMA. There are studies that aim to identify the primary condition between the two, the results on both sides, identifying a prevalence of 1.38% of celiac disease among lupus patients, as well as an apparent three times higher risk of SLE in people with CD (>20% presenting anti-ds-DNA, an aspect that raises the suspicion of the involvement of the microbiome in systemic autoimmune diseases) compared to the general population [33,34].

### 4.1. Genome-Wide Association

Although it is known that environmental stimuli can predispose individuals to the development of autoimmune diseases, studies focusing on related individuals and twins have shown that genetic factors as well as non-random inheritance of alleles (linkage disequilibrium) also play an important role in influencing the risk of their appearance [35,36]. The hypothesis that the presence of CD positively imprints the risk of SLE was confirmed by studying two samples with the help of Mendelian randomization (MR) based on the datasets related to the association at the genome level (GWAS) of different diseases involving the immune system [34]. In support of this information, we considered the identification of some groups of genetic risk factors, such as single nucleotide polymorphisms (SNPs), which are involved in the emergence and evolution of several autoimmunities manifested in the form of CD, Crohn’s disease, multiple sclerosis (MS), rheumatoid arthritis (RA), SLE, or type I diabetes (T1D). This is how the idea of common variants encountered in the pathogenesis of diseases is outlined, but it remains in debate whether the majority of genetic variants that contribute to complex traits of the population are caused by rare or frequent variants [35,37].

Many of the recently identified autoimmunity genetic locations (loci) at the chromosome level are shared between various autoimmune diseases, suggesting that subgroups of them are prone to showing similarities regarding the etiology and pathogenic mechanisms implicated. The degrees to which autoimmune disorders are characterized by shared (rather than unique) susceptibility loci vary substantially, from the entire number of shared loci (for RA) to 50% or more shared for CD, psoriasis, MS, SLE, T1D, ankylosing spondylitis, and autoimmune thyroid disease. Regarding the SLE-CD relationship, they seem to share loci such as TRAF1, IL12A, KIAA1109, and TNFAIP3, an aspect identified within two groups (out of a total of four identified) of associations of autoimmune diseases: group two that includes CD, RA and SLE and group four in which associations between T1D, RA, CD, Crohn’s disease and SLE are described [38].

Other genetic variations intensively studied regarding their association with autoimmune diseases, in particular, SLE, were the R620W polymorphism of the protein tyrosine phosphatase non-receptor type 22 gene (PTPN22), MYO9B, and CLEC16A (chromosomal position 16p13) [39,40,41]. Regarding the first one, there is contradictory evidence with reference to the increase in the genetic risk for CD [39,42,43]. Variations of the latter have also proven their involvement in the development of autoimmunity in celiac disease, CLEC16A being recognized for its impact on the phenomenon of immune tolerance resulting from thymic selection through autophagy of epithelial cells [41].

GWAS have therefore proven useful as a starting point in guiding and complementing functional studies, as they emphasize the importance and integrate the mechanisms of the processes involved in disease determination, indicating specific molecular pathways and cellular/organ models and offering a promising direction in the discovery of potential leads of targeted therapy [44].

### 4.2. The Impact of Viral Infection

Infections with enteric viruses (Coxsackie B and Rotavirus), influenza A, and herpesviruses can modulate the induction and development of autoimmune diseases while also playing a protective role, depending on several factors such as the genetic background, the host’s immune responses, the type of virus strain, viral load, and time of onset of infection. Viral-induced autoimmunity can be activated by several mechanisms, including molecular mimicry, epitope spreading, bystander activation, and immortalization of infected B cells, while protective effects can be achieved by activating regulatory immune responses, thus suppressing the development of autoimmune reactions. An important detail regarding herpesviruses is their persistence in the form of a latent infection that contributes to the pathogenesis of the systemic autoimmune disease at the time of reactivation [45].

The Epstein–Barr virus (EBV) is suspected of having a central role in autoimmune diseases, increasing the risk of SLE by up to 50% in children, an aspect supported by the increase in EBV DNA, mRNA, and titers of anti-early antigens IgG and IgA detected in their blood. The mode of action is based on molecular mimicry, inflammation, activation of innate immunity and production of type I IFN and pro-inflammatory cytokines, with immune evasion and anti-apoptosis. At the genetic level, the protein implicated in anchoring to loci with susceptibility to SLE, CD, or other autoimmunities is represented by EBNA2, together with transcription factors (numbering 20, of which at least 4 are therapeutic targets of current medication). A protective association between herpesvirus infections and celiac-type autoimmunity has also been shown, as evidenced by the inverse relationship between anti-CMV, EBV, and/or HSV-1 IgG levels and TGA. In addition to modulating the immune system toward Th2, viral infections, including CMV and EBV infections, can cause immune cell apoptosis, thereby dampening the immune system response and minimizing the progression of autoimmune diseases [45,46].

### 4.3. The Influence of the Internal and External Environment

#### 4.3.1. The Microbiome

In humans, the intestinal microbiota tends to stabilize and reach a greater diversity around the age of three years, influencing a multitude of physiological or pathological processes of the host either directly (at the digestive level) or remotely by creating links with vital organs such as the heart, working together to maintain homeostasis. In evolution, until adulthood, Gram-positive bacteria such as *Clostridium*, *Bifidobacterium*, *Lactobacillus*, *Ruminococcus*, *Streptococcus*, and Gram-negative bacteria such as *Bacteroides* and *Escherichia* appear in the intestine. Intestinal dysbiosis observed in autoimmune diseases is associated with a decrease in both bacterial function and diversity, damage to the intestinal barrier function, increased inflammation, and a decrease in regulatory T cells in the intestine, as well as, possibly, with molecular mimicry and T-cell activation, favoring a pro-inflammatory or posttranslational modification of luminal proteins. Worthy of discussion is the variable course of autoimmune diseases in each individual, including monozygotic twins, an observation that reiterates the contribution of environmental factors to the pathogenesis of the disease [47,48,49,50]. More and more studies are focused on the correlation between changes in the intestinal microbiota and the occurrence of autoimmune diseases, the main causal evidence regarding CD and SLE predominantly involving the bacterial genera *Bifidobacterium* and *Ruminococcus*. Thus, we identified a lower risk of developing SLE, correlated with a higher risk of CD, in people with a high level of *Bifidobacterium*, together with a higher risk of SLE, but a negative association of CD with respect to *Ruminococcus*, and a decrease in the *Firmicutes/Bacteroidetes* ratio in SLE. It therefore remains open to debate the possible beneficial influence or not of probiotic treatment, with *Bifidobacterium* among children with CD, depending on the type of strain used [51].

#### 4.3.2. Atopy

The association between allergic diseases and autoimmunity is still being researched; a retrospective cohort study carried out in 1990–2018 that included patients of all age categories, doubled by a transversal study, concluded that the risks of developing of autoimmunity (including CD and SLE), in the long term are increased in subjects with atopic terrain (allergic rhinitis/conjunctivitis, atopic eczema, asthma), being distributed in groups according to age and gender [52].

#### 4.3.3. Vitamin D Deficiency

It has been shown that immune cells, including dendritic cells, macrophages, and T and B cells, express the vitamin D receptor and 1α-hydroxylase, substances with a role in both calcium homeostasis and the mineralization of the collagen matrix, as well as immunomodulators in innate and adaptive immunity (through control of immune cell growth and differentiation), being anti-inflammatory, antioxidant, and anti-fibrotic. Another known effect of vitamin D is maintaining the integrity of the intestinal barrier (essential in preventing dysbiosis) by regulating the colonic mucus, influencing the composition and functions of the intestinal microbiota, and modulating the release of zonulin. Regarding the intake of vitamin D administered daily (in case of minimal exposure to the sun), the consensus was reached that it represents 400 IU/day for ages older than 1 year, 600 IU/day for ages between 1 and 70 years, and 800 IU/day for 71 years and older, while the upper tolerable level varies between 1000 and 4000 IU/day, comprising 1000 IU for infants 0–6 months, 1500 IU infants 6–12 months, 2500 IU for children 1–3 years, 3000 IU for children 4–8 years, and 4000 IU for children 9 years and older. The prevalence of insufficiency (21–29 ng/mL or 52–72 nmol/L) and vitamin D deficiency (below 20 ng/mL or 50 nmol/L) were also studied among children, recording values of 61% and 9%, respectively, in a study group made up of 6275 participants, with data proving that vitamin D supplementation for five years, with or without omega 3 fatty acids, can reduce the risk of autoimmune diseases by 22%. The association between vitamin D and autoimmune disease has also been observed to be subject to seasonal variation (higher prevalence in spring-born children) as well as latitude (higher prevalence in northern countries with less UVB radiation) [53,54,55,56].

With reference to SLE and CD, most of the existing studies in the literature emphasize that the low level of 25 (OH) D in the patient’s serum (easier to identify among adults who, compared to children, do not receive food enriched with vitamin D in order to prevent rickets that can mask this characteristic), in the absence of another obvious cause, must raise the clinician’s suspicion of overlapping autoimmunity, especially because of the solid evidence regarding its involvement in the pathogenesis of the disease. Aspects worthy of emphasis regarding the two examples of diseases are the objectives of reducing SLE activity and protecting against lupus nephritis induced by vitamin D treatment, but also the improvement of vitamin D levels following the gluten-free diet established among CD patients [55,57,58]. In conclusion, there is an important correlation between vitamin D and autoimmunity, an aspect that reveals the need to know the bidirectional relationship between the two entities, the importance of screening for SLE and CD among people with unexplained deficits, vitamin D dosage in case of unsatisfactory response to therapy, and the establishment of the appropriate supplement.

#### 4.3.4. Prolactin Level

Prolactin (PLR) is secreted both by the anterior pituitary gland and by various extra-pituitary sites (including immune cells); hyperprolactinemia (>18 ng/mL in boys and >24 ng/mL in girls) is therefore described in the phases of active autoimmune diseases, including SLE and CD, as the stage in which it determines increased synthesis of IFN-gamma and IL-2 by Th1 lymphocytes and the activation of Th2 lymphocytes with consecutive production of autoantibodies, correlating with the age of diagnosis, the duration of symptoms, the degree of villous atrophy, and infiltration of the lamina propria [59,60]. Regarding CD, it seems that hormone levels are directly related to the gluten-free diet, registering a decline in PLR, simultaneously with the anti-transglutaminase antibodies, after six months of a gluten-free diet. In SLE, in addition to the impact on serology, PRL seems to leave a mark on neurological, renal, and hematological function, as well as the involvement of serous cells in the clinic of the disease [61]. A positive aspect for young girls resides in the fact that CD does not seem to affect the ovarian reserve, an aspect confirmed by tracking its markers (antral follicle count, ovarian volume, and anti-Müllerian hormone) in a group of 45 teenage girls on days 2–5 of the menstrual cycle [62]. Anti-prolactin autoantibodies were also identified in SLE patients, considering that all SLE patients with anti-PRL autoantibodies had hyperprolactinemia (hPRL) and only 31.7% of SLE patients classified with idiopathic hPRL had anti-prolactin antibodies. Subjects with idiopathic hPRL and anti-PRL autoantibodies have less clinical and serological activity of SLE and do not experience the classic symptoms of hyperprolactinemia such as menstrual disturbances and/or galactorrhea [63].

#### 4.3.5. The Overwork

Chronic, deep fatigue, defined as periods of debilitating exhaustion that interfere with normal activities by decreasing concentration, orientation, and mental performance, is also one of the frequently encountered symptoms. Given the role played by inflammation through various mechanisms (starting from pro-inflammatory cytokines such as IL-1β, TNF-α, IL-6, and IFN-γ) in inducing fatigue, inflammatory pathways and subsequent physiological changes are considered potential treatable targets in patients with autoimmune disease. Moreover, the association of comorbidities such as anxiety, depression, or pain complicates the burden on patients. A bidirectional relationship seems to exist between insomnia, circadian disorders, increased sleep apnea index (in SLE), and autoimmunity. Consequently, immunomodulatory agents and drugs targeting inflammatory pathways can serve to treat fatigue and enhance quality of life [64].

#### 4.3.6. The Psychic Component

In agreement with the previous statements, an important element that contributes to the appearance of fatigue is direct or indirect damage to the central nervous system [64]. Psychological stress, including social stress, also seems to be a risk factor in the alteration of the intestinal barrier (consisting of a layer of mucus, intestinal epithelial cells, tight junctions, immune cells, and intestinal microbiota), outlining a vicious circle together with the psychological impact determined by the disease, which can either trigger or aggravate autoimmune manifestations in children [65,66,67]. In addition, a longitudinal cohort study, which included 2.192.490 children together with their parents, concluded that autoimmune disease was more frequent among children and adolescents with a parental history of autoimmunity (respectively, 7.1% compared to 4.3%) and in girls (54.3% versus 45.7%), with little evidence of an association between autoimmune diseases and most parental mental disorders (e.g., reduced risk for CD identified among children with maternal non-psychotic affective disorders). This finding is delimited by the data in the literature regarding the increased risk of CD among first-degree relatives (parents/siblings) of people with schizophrenia [68]. An important role in the health of these patients is played by the practice of physical activity, which has known effects in terms of both reducing stress and improving cognitive status, but has recently shown a positive impact on the homeostasis of the immune system by modulating the number and function of immune cells, inhibiting the body’s systemic inflammatory response, and delaying the onset and development of autoimmune diseases [69,70].

### 4.4. Consequences of IgA Deficiency

Selective IgA deficiency (SIgAD), the most common primary immunodeficiency in the Western world (with a prevalence of approximately 1:600 in the general population and the possibility of progression to variable common immunodeficiency of 23.5% among subjects with autoimmunity), is a disease with polygenic determinants that, in part, can overlap with genes associated with autoimmune diseases. The diagnosis is confirmed by the association of immunoglobulin A (IgA) levels lower than 0.07 g/L manifested among patients over four years old, with immunoglobulin M (IgM) and immunoglobulin G (IgG) within normal limits and without any other cause identified for the immunodeficiency. Because immunoglobulin is strongly expressed at the level of the mucous membranes, its deficiency is marked by recurrent infections, allergic disorders, and autoimmune manifestations; therefore, screening is essential in these circumstances [71,72,73].

Numerous studies have identified both systemic and organ-specific autoimmune disease in SIgAD, with an incidence between 3% and 79% (depending on ethnicity and condition) and a double predisposition among first-degree relatives of people with SIgAD, compared to the general population, an aspect attributed to the decrease in the defense of the gastrointestinal mucosa and the increase in humoral and cellular interactions. Not infrequently, autoantibodies were identified in the patient’s serum even in the absence of specific symptoms [74].

From the variety of autoimmunities encountered in SIgAD, possibly because of the specific haplotypes of the human leukocyte antigen (HLA-A1, B8, DR3, and DQ2) associated with the two conditions, we will address SLE and CD in the following. Thus, studies indicated a frequency of SIgAD among subjects with SLE and CD of 1:19–130 (prevalence > 30 times compared to the general population) and 1:192 (prevalence > 5–15 times compared to the general population), respectively [75]. The strong association between SIgAD and celiac disease complicates clinical practice both by the fact that most laboratories use IgA-based tests (anti-tissue transglutaminase and/or anti-endomysial antibodies) for the diagnosis of CD and by the low susceptibility of these individuals to manifest gastrointestinal symptoms, inversely correlated with the risk of underdiagnosis, but without clear evidence of the impact of untreated CD on the incidence of lymphomas. Therefore, it is important to obtain a total IgA level along with these tests, as there may be false negative serological tests in the presence of SIgAD [73,74]. With regard to SLE, an increase in the probability of the association of anti-Sm, anti-La, and ANA was found among those with concomitant SIgAD, without influencing the symptoms, the titer of anti-Ro antibodies, and the HLA [73].

### 4.5. The Place of Nutrients in Pathogenesis

With birth, a complex process is started, a process that affects the mother as well as the fetus (depending on the time and the circumstances in which it was born). The latter goes through an intense period of adaptation to environmental factors during childhood and adolescence, an adaptation that leaves its mark on their health and the balance of later adult life [76]. All of these marks can potentiate the occurrence of autoimmune diseases, a group of diseases which, although considered heterogeneous, have many common aspects from a pathogenic and evolutionary point of view, aspects emphasized with the help of the celiac disease–lupus autoimmune association model. Studies regarding the involvement of diet in the modulation of the immune system are in continuous development. The first argument for this theory is represented by the observation of an increase in the incidence of these types of diseases, especially in Western countries, possibly in association with the great diversity of diets and unhealthy eating habits based on high contents of fat, sugars, and total calories added, in contrast to a low fiber content (which is accentuated by its inclusion in a gluten-free diet for children with CD) and an imbalance in the use of fatty acids. These mistakes can promote the alteration of the intestinal barrier function, together with the decrease in the diversity of the microbiome, directly proportional to the diversity of the diet [77,78,79,80].

The mechanism by which dietary fibers influence the intestinal barrier and the immune system involves their fermentation by intestinal bacteria, with the subsequent production of short-chain fatty acids. These represent both an energy substrate for intestinal cells and a pawn in the development and differentiation of regulatory T cells, thus strengthening beliefs about the diet–microbiota–immunity triad [81]. Other food factors whose balance influences the pathological process (excluding vitamin D, already discussed) are vitamin A, vitamin E, selenium, calcium, iron, magnesium, zinc, omega-3 fatty acids, phytoestrogens, and flavanols, compounds that seem to influence at the same time regulatory T cells and cytokine production and also the development of extra-intestinal manifestations from the neurological, psychiatric or locomotor system spheres. Thus, their supplementation is beneficial for increasing life expectancy and its quality [80,82,83,84,85,86].

At the same time, numerous studies present in the literature highlighted the impact played by epigenetic changes such as DNA methylation (hypomethylation) or histone acetylation in the onset and evolution of SLE. The substances with a beneficial role in influencing this process proved to be folic acid, methionine, choline, and some vitamins from the B complex (methyl donor nutrients) [87].

Reviewing the most important aspects of each food constituent involved in regulating the compensation–decompensation balance of autoimmunity, we observed the following:

Fats. Their consumption is misleading, especially among CD patients, where it seems that lipids are the main constituents of gluten-free products (bread and pasta). Another unfortunate aspect regarding the consumption of lipids is represented by the incorrect dosage of the ratio of saturated fatty acids versus unsaturated fatty acids in the diet [80].

Fiber (required 25–31 g/day). There are components that seem to be in deficit both in children under a gluten exclusion regime and in children with a normal diet (possibly also because of the tendency toward a Western diet, excluding foods rich in fiber in favor of processed ones), but being more accentuated in the first case. Results have led to increased interest in alternating gluten-free diets by including compounds such as quinoa, buckwheat, and amaranth [77,80].

Fatty acids. Omega-3 fatty acids (eicosapentaenoic and docosahexaenoic) represent substrates for the synthesis of signaling molecules, also exhibiting immunomodulating action on lymphocytes, cytotoxic T cells, natural killer cells, macrophages, monocytes, and neutrophils. It is well known that diet is the main source of essential fatty acids (linoleic and alpha-linoleic) [77,82].

Vitamins:

- A: having as its origin pro-VitA, retinol, or retinyl ester, vitamin A in insufficient concentrations deregulates the function of regulatory T cells, causing an excess of T helper 1 in favor of T helper 2, an observation that sparked interest in the study of all-trans retinoic acid (ATRA), the main metabolite of vitamin A [85];
- B Complex: represents one of the lines of micronutrients affected in autoimmune diseases, and it is therefore essential to know the inverse correlation between the level of total homocysteine in the plasma and that of the vitamins in the B complex (B1, B2, B6, B9), a marker that also proved its effectiveness in a study of the quality of life among adult patients with CD [80].

Minerals. One in ten patients with CD suffer from mineral deficiencies, the gender ratio being equal in terms of calcium and magnesium, while zinc and iron (influenced by factors such as the severity of villous atrophy and the degree of fortification of wheat flour) are deficient in men and women, respectively. Aspects worthy of the clinician’s attention regarding the evolution of subjects with CD, especially children, are the increased frequency of the association between iron deficiency anemia and CD; the role played by zinc in protein synthesis, thus imprinting the body’s growth, the inflammatory response (Il-2 and Il-2 receptor alpha), and cellular changes; but also the life-threatening consequences of copper deficiency, affecting numerous processes such as the synthesis of hemoglobin and neurotransmitters, iron oxidation, cellular respiration, the formation of pigments and connective tissue [80,82].

Flavanols (4.2 mg–3 g/day). They interact with the pro-inflammatory cytokines of type Il-1 beta and Il-2, with TGF-beta1 and TNF-alpha, the beneficial effects being best demonstrated by the study of spirulina, known for its content rich in flavanols and sulfalipids [82].

Phytoestrogens. They influence both humoral and cellular immunity by acting on IL-2, T helper 1 and T helper 2 lymphocytes, but also on the inflammatory response, modulating the body’s reaction to autoantigens [83].

Methionine and Choline. Two compounds involved in the synthesis of S-adenosyl-methionine (SAM), thus being involved in the DNA methylation process that can affect the expression of genes involved in the immune response, with still uncertain recommendations regarding supplementation among patients with SLE (mainly methionine, owing to side effects) [87].

**Figure 1:**
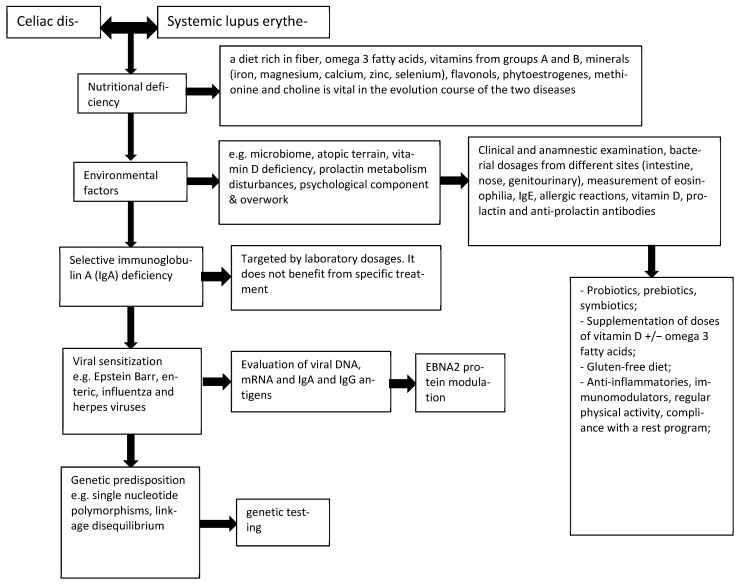
Diagnostic algorithm of complications associated with SLE-CD.

Taking into account the variety of aspects that interconnect SLE with CD, there was a need to form an algorithm based on diagnostic criteria for the affected population with the aim of improving the quality of life by recognizing and counteracting treatable conditions (Figure 1).

## 5. The Practical Component

In the literature there are numerous examples of the coexistence and interactions of autoimmune diseases, aspects that will be exemplified in the following with an emphasis on SLE and CD. In this sense, we must also consider the existence of gluten sensitivity apparently manifested in the form of SLE with extra-intestinal signs such as IgA deficiency, increased double-stranded DNA (in the absence of antinuclear factor), increased inflammatory markers, and symptoms suggestive of an immune diathesis in the absence of an enteropathy [88]. Systematizing the published data, Table 2 shows the overlap between the two conditions among children.

## 6. Treatment

As two autoimmune diseases that share many overlaps in terms of pathogenesis and clinical practice, SLE and CD also follow similar general lines in therapeutic management that include the avoidance of triggering factors, the use of steroid agents in the treatment of the acute phase, and the induction of symptomatology remission and pharmaceutical preparations directed against the various stages of the pathogenic cascade, as presented in Table 3.

Except for the common model of the development and evaluation of autoimmune diseases, previously developed with the help of the SLE-CD correlation, they seem to be interconnected at the therapeutic level as well. Recent studies are contradictory regarding the identification of the impact obtained by the introduction of a gluten-free diet among patients with autoimmune comorbidities such as thyroiditis and type 1 diabetes, without being able to eliminate the risk of bias. The possible positive causal link between the establishment of a gluten-free diet in the context of celiac disease and the evolutionary course of other associated autoimmunities is therefore emphasized; in order to define it more accurately, further studies are needed [96,97,98].

Therefore, new paradigms are introduced regarding the control of autoimmune diseases with the help of balanced, carefully chosen diets. Starting from the premise “we are what we eat”, we demonstrated in the previous sections that, although there are multiple links at the level of the etiological factors determining autoimmune diseases and the clinical aspects (which can sometimes go as far as overlapping pathologies), one of the most consistent roles in the control of the condition is played by dietary constituents. This idea must be considered by the clinician and exploited in the patient’s therapeutic course, it being an easy treatment measure with a low cost and psychological impact, but with numerous benefits in increasing the patient’s quality of life.

Continuing the CD-SLE model, we discuss a list of foods in the composition of which high concentrations of the most important nutrients in autoimmune diseases are found:

- Folic acid: green vegetables, bell peppers, beans, lentils;
- Methionine: eggs, yogurt, cheese, red meat;
- Choline: beef liver, egg, soy, potatoes, quinoa, peanuts, carrots, apples, broccoli;
- B complex vitamins: rice, quinoa, apple, strawberries, bananas, watermelon, walnuts, spinach, onions, tomatoes, chickpeas, beans, potatoes, salmon, tuna, beef liver, milk, yogurt, cheese;
- Flavanols: cocoa, red grapes, tea, berries, apples;
- Vitamin A: liver, carrots, sweet potatoes;
- Vitamin E: quinoa, amaranth;
- Omega-3 fatty acids: chia/flax seeds, sardines, milk, beans, salmon, soy [80,82,85,87,99].

In addition to the challenges of everyday life, what can “induce/discover” or leave a negative imprint on the already known pathologies (remembering here the recent pandemic with the SARS-CoV-2 virus that we dealt with, marking the evolutionary course of both autoimmunity and other nutritional or metabolic disorders), a modern approach in terms of immunity is represented by the study of intestinal dysbiosis, particularly the use of probiotics, with known effects in the case of atopies, but still in studies for autoimmune diseases, the hypothesis being advanced that small doses of probiotics could have be more effective immunomodulators, compared to high doses [82,100,101]. The mechanisms of action by which they modify the composition and function of the intestinal mucosa, the microbiota, and also the immune system are represented by encouraging the development of beneficial bacteria (*Firmicutes, Bifidobacteria*), at the expense of harmful ones (*Enteroccocus*), creating a pro-tolerogenic environment and modulating pro-inflammatory responses by acting on innate and adaptive immunity [85,102].

## 7. Conclusions

In conclusion, intensively studied and still incompletely elucidated, the SLE-CD relationship is worthy of consideration in clinical practice as representing a model of the multiple interactions of autoimmunity. The current work presented the interactions between the two pathologies from the perspective of their common aspects, starting from similar diagnostic models (presence of autoantibodies, circulating immune complexes, inflammation and multisystem damage, with histological impact on the target organs), the influence at the genomic level, the impact of viral infections and IgA deficiency, the endogenous and exogenous factors that modulate the clinical expression, and also the common therapeutic lines in acute and long-term management. It was thus emphasized that the two pathologies are strongly correlated on multiple levels; there are also presented in the literature cases of the overlap of these, with or without other autoimmune diseases, which draws attention to the way in which they influence each other. The question that remains, arousing the interest of future studies, is “Which of them was the first?”, while the factors that can anticipate the development of the afflictions, thus worsening the prognosis, are not to be neglected either. We thus conclude by reiterating the fact that a good knowledge of the many interactions that occur at the level of the autoimmune system is important in future medical practice, as the evolution of treatment means must go in tandem with the evolution of prophylaxis means in order to maintain an optimal quality of life.

## Figures and Tables

**Table 1:** Diagnosis of CD and SLE (adapted from Al-Toma A et al., Ameer MA et al., Ribero S et al., Almaani S et al., Fanouriakis A et al., Lecouffe-Desprets M et al. and Al-Mogairen SM et al.) [17,18,19,20,21,22,23].

	**Diagnostic Investigations**
Celiac disease	Serological: AGA: used in the past, with 85% sensitivity and 90% specificity; TGA and EMA: tested using ELISA and less commonly on RIA; -IgA-EMA can be used for confirmation when TGA has a low titer (<2 times the upper limit of normal), although biopsy is indicated. ! anti-TGA test is the most sensitive test for CD, whereas IgA-EMA is the most specific test; IgA and IgG-DGP: can be almost as sensitive and specific as IgA-TGA, albeit more expensive; IgA deficiency.
	Endoscopy and biopsy: Approximately one third of newly diagnosed cases of CD have a completely normal endoscopic appearance, the biopsy of at least four quadrants being therefore mandatory, following aspects such as mucosal fissuring, nodular mucosa (mosaicism), bulb atrophy with visible submucosal vessels and loss, and reduction or scalloping of Kerckring folds.
	Modern diagnostic methods: Flow cytometry of scattered IEL: diagnostic specificity up to 97%; Test for HLA-DQ–gluten tetramer in blood for detection of gluten-specific CD4 + T cells: not available outside the research setting; IFN-γ-secreting T cells reactive to gluten: using the ELISPOT assays or by flow cytometry tetramer technology; HLA-DQ2/8 typing: positive predictive value > 99%.
	Other investigations: Video capsule endoscopy: has better overall sensitivity for detection of macroscopic features of atrophy and the associated complications (stenosis, erosions, ulcers, and lymphoma); Intestinal permeability tests; * are not recommended for diagnosis; I-FABP: useful in identifying dietary non-adherence and unintentional gluten intake; Radiology: a decreased number of jejunal folds, an increased number of ileal folds, small-bowel dilatation, wall thickening, intussusception, mesenteric lymphadenopathy, vascular changes, and splenic atrophy; The gluten challenge test (10 g/day for 6–8 weeks): for the diagnosis of CD already on a gluten-free diet.
Systemic lupus erythematosus	Serological: ANA: identified with IF/ELISA/multiplex assays; anti-dsDNA: correlated with renal involvement by their deposition in glomeruli, basement membrane, and mesangium in SLE patients with active nephritis; anti-Sm: It correlates with disease activity, showing a relatively static expression in peripheral blood (unlike anti-dsDNA antibodies, which are fluctuating during the course of the disease), and an association with lupus nephritis; SSA and SSB: useful to evaluate secondary Sjögren’s syndrome in patients with SLE, subacute cutaneous lupus, photosensitivity, and neonatal lupus; Complement C3 and C4: when used in isolation, their reliability as biomarkers for SLE can be limited.
	Endoscopy and biopsy: association between eosinophilic gastrointestinal disease and autoimmune diseases of the connective tissue (including SLE); the existence of an SLE-associated protein-losing enteropathy (manifested as edema of the intestinal mucosa, together with atrophy, vasculitis, infiltrate with inflammatory cells and lymphangiectasia); therefore, it is important to use biopsy in order to exclude it or esophagitis, gastritis, or even eosinophilic enteritis.
	Biopsy of the main affected organs: Skin: typical pathological findings include basal and suprabasal keratinocyte vacuolization and necrosis, basal membrane thickening, pigment incontinence, and a cellular infiltrate at the epidermal/dermal junction (neutrophils/lymphocytes); Kidneys: mesangial, subendothelial or subepithelial immune complexes, proliferation of mesangial cells, segmental or global endocapillary proliferation, glomerulosclerosis.
	Other investigations: urine sample; blood gas analysis; blood count, peripheral blood smear, Coombs test; thoracic and joint radiography; functional brain MRI; Cardiac MRI and highly sensitive troponin testing.

* AGA: IgA-anti-gliadin antibodies, ELISA: enzyme-linked immunosorbent assay, RIA: radioimmunoassay, DGP: deamidated gliadin peptides, IEL: intraepithelial lymphocytes, IFN: interferon, ELISPOT: enzyme-linked immunospot, I-FABP: intestinal fatty acid binding protein, IF: immunofluorescence assay, anti-Sm: Anti-Smith antibodies, SSA: Anti-Ro antibodies, SSB: Anti-La antibodies.

**Table 2:** CD-SLE interests in clinical practice (adapted from Zitouni M et al., Naseem S et al., Calvani M Jr et al. and Latif S et al.) [89,90,91,92].

Conditions	Characteristics of the Cases	Reference
SLE with concurrently occurring CD, before SLE and postSLE	Retrospective study of 5 cases encountered within 4 years, including one child with CD before the onset of SLE. CD was histologically confirmed by the identification of villous atrophy on the duodenal biopsy fragment, with favorable evolution when gluten was excluded. SLE criteria were also fulfilled, the tests for anti-nuclear and anti-dsDNA antibodies being positive, and C3 and C4 levels low.	Zitouni M. et al. [89]
Anticoagulant lupus associated with CD	Female child, 8 years old, showing weakness, abdominal discomfort, unsatisfactory weight curve and bullous lesions (which spared the face) for about 3 months. Paraclinical in evolution: - anemia (hemoglobin-68 g/L); - G6PD deficiency; - eosinophilia and nutritional anemia for iron (Perls stain was 0, which is equivalent to an iron content of 42 ± 23 lg/g); - vitamin B12 folate deficiency (megaloblastic hematopoiesis); - partial villous atrophy on duodenal biopsy; - raised tissue transglutaminase levels (13 U/L; C B 5 U/L); - extensive inflammation of muscles and fascia on magnetic resonance imaging of left leg; - APTT—54 s (control 36–45 s) with positivity for LA by both KCT and dRVVT; - HLA-DR3 positive.	Naseem S. et al. [90]
Latent CD in the presence of epilepsy and cerebral calcifications, with drug-induced SLE and malabsorption of intestinal folic acid	A 15-year-old male patient under treatment with valproic acid, ethyl phenylbarbiturate, and ethosuximide presented the suspicion of drug-induced SLE. Paraclinical in evolution: - AGA and EMA: initial EMA titer 1:50, with dynamic increase to 1:200; - intense chronic inflammation without villous atrophy or cryptic hyperplasia (biopsy I); villous atrophy (biopsy II, after 15 months); - folic acid: 1.8–2.0 ng/mL, with the decrease to 11.8 ng/mL following the exclusion of gluten; - impaired intestinal absorption of folic acid; - defect in crossing the blood–brain barrier; - folic acid in CSF: 12.6–13.9 ng/mL (limits 15–40 ng/mL), together with the change in the CSF/serum folic acid ratio.	Calvani Jr M et al. [91]
Multiple autoimmune syndrome: Hashimoto’s thyroiditis, CD and SLE	Female patient, aged 11 years, overweight, pale, with clinical swelling in front of the neck (5 × 8 cm) along with constipation, anorexia, weight gain, and increasing pallor over a period of six months, symptoms associated with episodes of joint (knee and ankle) and abdominal pains in the antecedents, which could not be correlated with diet or other symptoms from the gastrointestinal or genitourinary, sphere. Paraclinical in evolution: - hemoglobin: 5.6 gm/dL (limits 10–11.5 gm/dL); - microcytic hypochromic anemia; - serum ferritin: 4.92 ng/mL (limits 20–200 ng/mL); - increased TSH: 13.82 units/mL (normal 0.17–4.05 units/mL); - low T4: 0.72 g/100 mL (range 0.89–1.79 g/100 mL); - normal T3: 3.60 ng/mL (limits 1.62–3.77 ng/mL); - serum thyroglobulin antibodies and antiperoxidase antibodies positive: the values of 1:160 (1:10 titer) and 1:400 (1:100), respectively; - ANA and anti-ds-DNA positive; - C3, C4 within normal limits; - TGA-IgA positive: 21.1 U/mL (limits: 0–7); - intestinal biopsy with appearance of subtotal villous atrophy and chronic inflammation.	Latif S. et al. [92]

TSH: thyroid-stimulating hormone, G6PD: glucose 6-phosphate dehydrogenase, APTT: prolongation of activated partial thromboplastin time, LA: lupus anticoagulant, KCT: kaolin clotting time, dRVVT: dilute Russell viper venom time, AGA: positive anti-gliadin.

**Table 3:** General directions in the therapeutic management of SLE and CD (adapted from Al-Toma A et al., Fanouriakis A et al., Rubio-Tapia A et al., Pan L et al., and Fanouriakis A et al.) [17,21,93,94,95].

Acute hives	**Celiac disease**	**Systemic lupus erythematosus**
	Hospitalization and parenteral nutrition—in celiac crisis (risk of hemodynamic instability, orthostatic hypotension, neurological and renal dysfunction, metabolic acidosis, hypoalbuminemia, and electrolyte disorders); Steroids;	Hospitalization and rebalancing of renal, hematological, neuropsychological, and other affected organ systems; Pulsations with intravenous methylprednisolone (250–1000 mg/day for 3 days), doses adapted according to severity and body weight, administered after excluding infections;
Background treatment	• Avoiding the factors that precipitate the disease: gluten-free diet (safety limit between 10–100 mg/day), with the elimination of cereals and food products derived from wheat, barley, or rye; Supplying nutritional deficiencies: iron, calcium, copper, zinc, folate, vitamin B12, B6, D; Fiber-based diet (corn, potatoes, vegetables); For refractory CD: - Steroids-Oral Budesonide/Azathioprine (2–2.5 mg/kg/day) + Prednisone; - Infliximab; - Mesalamine; - Purine analogue inhibitors (Cladribine or Fludarabine): 0.15 mg/kg/day for 5 days; - Jak3 inhibitor (Tofacitinib) or anti-Il-15; - Transplantation of autologous hematopoietic stem cells.	Avoiding exposure to ultraviolet radiation, smoking and drugs with the potential to induce SLE; Hydroxychloroquine (<5 mg/kg)—presents a risk of retinopathy; Glucocorticoids (<7.5 mg/day); Cyclophosphamide—may increase the risk of malignancies; Immunomodulatory agents (Methotrexate, Azathioprine, Mycophenolate); Belimumab; Rituximab (anti-CD20)—with response on refractory SLE; Tocilizumab (anti-Il-6); Etanercept (anti-TNF).

## Data Availability

Not applicable.
